# Ensemble modeling uncovers climate change-driven distribution shifts of *Fargesia nitida* (Mitford) P. C. Keng ex T. P. Yi, a primary food source for giant panda

**DOI:** 10.3389/fpls.2025.1683441

**Published:** 2025-12-01

**Authors:** Ling Mao, Chunling Wang, Shanshan He, Feng Deng, Xiaying Ye

**Affiliations:** Agronomy and Life Science Department, Zhaotong University, Zhaotong, Yunnan, China

**Keywords:** alpine bamboo, Biomod2, climate change, conservation, distribution pattern, *Fargesia nitida*, giant panda

## Abstract

*Fargesia nitida* (Mitford) P. C. Keng ex T. P. Yi is an alpine bamboo species endemic to the Hengduan Mountains (HDM). As the primary food source for giant pandas and a key component of subalpine ecosystems, it plays an irreplaceable ecological role. However, its exceptionally long flowering cycle (up to 109 years) and limited dispersal ability render it highly vulnerable to climate change impacts, while the potential shifts in its distribution under future climate scenarios remain inadequately explored. In this study, we employed ensemble modeling (Biomod2) to project the suitable habitats under current and future climate scenarios (SSP126, SSP245, SSP585) for the 2050s and 2090s, based on 78 occurrence records and seven environmental variables. Results showed that the ensemble model exhibits superior predictive performance (AUC = 0.995, TSS = 0.957, Kappa = 0.753) compared to optimized MaxEnt and other individual models. Temperature seasonality (bio4) and minimum temperature of the coldest month (bio6) were identified as the most influential factors. Currently, the total suitable habitat area is estimated at 83.10 × 10^4^ km², with a central distribution located primarily in the HDM region and its adjacent areas. Although future projections suggest an overall expansion of the total suitable area, the highly suitable habitats—corresponding to the species’ current occurrence area—show a persistent contraction. By the 2090s, this contraction will exceed 56% under the SSP585 scenario. Distribution centroid analyses revealed that the current center of *F. nitida* is located in northern Sichuan. It will shift northward to higher latitudes under the low emission scenario, but southwestward to higher altitudes under the high emission scenario. These findings underscore the vulnerability of *F. nitida* to climate change and provide critical scientific insights for the development of targeted conservation strategies, as well as for the effective management of giant panda habitats and subalpine ecosystems under future climatic conditions.

## 1 Introduction

Climate change, characterized by rising temperatures and altered precipitation patterns, poses a severe threat to global biodiversity (Arneth et al., 2020; Wudu et al., 2023), especially for mountainous biodiversity hotspots like the Hengduan Mountains (HDM). As a core hotspot in southwestern China, the HDM harbors over 3500 endemic seed plant species, accounting for approximately 29% of China’s total endemic seed flora (Mittermeier et al., 2004). However, these specialized species are confronting heightened risks from rapid climate shifts (He et al., 2019). Projections have indicated that under a warming scenario of 3.2 °C above the pre-industrial levels by 2100, about 49% of insects, 44% of plants, and 26% of vertebrates are expected to lose more than half of their historical geographic ranges (Warren et al., 2018). This loss will be further compounded by cascading extinctions and reduced ecosystem stability, exacerbating overall vulnerability (Koh et al., 2004; Correia and Lopes, 2023). Thus, predicting the future climate suitability for these endemic species in the HDM is critical for guiding targeted conservation efforts.

Climate change reduces the fitness of species by altering phenological patterns, disrupting interspecific interactions, and shifting climatic niches, thereby driving population declines and ultimately increasing extinction risks (Bellard et al., 2012). Species with restricted distributions and slower migration rates are especially susceptible to these impacts (Zhang et al., 2024; Alves-Ferreira et al., 2025), and bamboos (Poaceae: Bambusoideae) are a typical example. As the only major grass lineage diversified in forests, bamboos offer important environmental, social and economic benefits, but their exceptionally long reproduction intervals (up to 120 years) and short rhizomes (as short as 1 cm) limited dispersal capacity, leading to geographically constrained distributions—particularly among sympodial bamboo species (Janzen, 1976; Li et al., 2006). These characteristics prevent bamboos from keeping pace with the rapidly shifting climate envelopes, leaving them exposed to threats posed by accelerating climate change (Li et al., 2019; Zhao et al., 2023; Wang et al., 2024). Therefore, accurately evaluating climate impacts on bamboo species is critical for formulating effective conservation and development strategies— especially for the alpine bamboo genus *Fargesia* Franchet. Globally, there are ca. 90 *Fargesia* species, 86% (77 species) of which are endemic to China, with 67% (60 species) concentrated in the HDM (Li et al., 2006; Ye et al., 2019).

**Figure 1:**
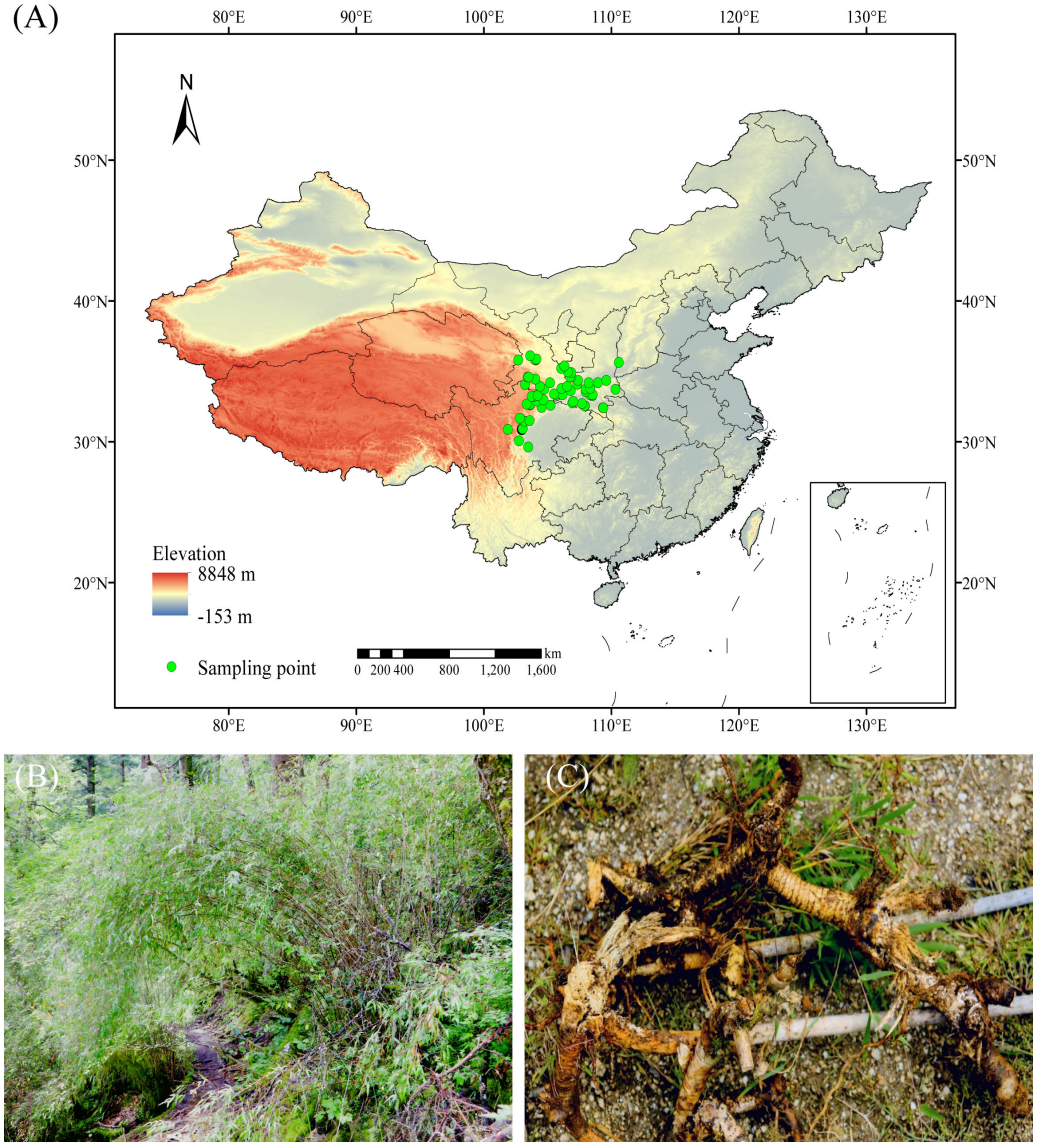
Fargesia nitida. **(A)** Sampling site; **(B)** Habitat; **(C)** Rhizome.

*Fargesia nitida* (Mitford) P. C. Keng ex T. P. Yi is an HDM-endemic understory bamboo species at an altitude of 2450 m to 3200 m. It possesses a typical caespitose sympodial rhizome (rhizome neck 10–13 cm, **Figure 1**) and an unusual extended flowering cycle, with intervals reaching up to 109 years (Ma et al., 2017). Furthermore, this species constitutes approximately 90% of the giant panda’s (*Ailuropoda melanoleuca*, IUCN Vulnerable) diet (Yi and Jiang, 2010). Its decline—driven by altered precipitation and temperature—threatens both pandas and subalpine forests. For example, historical records show that more than 200 giant pandas perished due to food scarcity during its flowering-induced mortality event in the 1980s (Yi et al., 2008). Molecular dating analyses of alpine bamboos indicate that *F. nitida* originated during the late Pleistocene and the *Fargesia* genus has likely suffered a decline in diversification since approximately 3 million years ago (Ye et al., 2019). This pattern suggests that climate change has long exerted negative effects on the species’ growth and evolutionary trajectory. While existing studies on *F. nitida* have primarily focused on its ecological characteristics and its relationship with giant panda (Wang et al., 2009; Guo et al., 2023; Shi et al., 2025), systematic analyses of its distribution pattern under future climate change scenarios remain limited.

Species distribution models (SDMs) have become essential tools for evaluating the impacts of climate change on species distribution and are widely applied in the field of conservation biology (Guisan and Zimmermann, 2000; Chen et al., 2025). These models project the potential species distributions and the associated ecological needs based on existing distribution data and relevant environmental variables (Srivastava et al., 2019). Currently, more than ten models have been developed, such as random forest (RF), generalized additive model (GAM), and the maximum entropy model (MaxEnt). Most studies employed individual model to forecast species distribution pattern under future climate scenarios (Santini et al., 2021). However, different SDMs are established based on distinct algorithmic principles and thus exhibit unique strengths and limitations when applied to the same species (Bellard et al., 2012; Qiao et al., 2019). The Biomod2 package, an ensemble modeling technique that integrates multiple models, is regarded as an effective approach to enhance accuracy and optimize predictive results (Yang et al., 2024; Kang et al., 2025). Meantime, area under the curve (AUC) of the receiver operating characteristic (ROC) curve, true skill statistics (TSS), and Kappa values can be used to assign weights to each model, yielding the best simulation outcomes.

In this study, we focus on *F. nitida*, the primarily food source of the giant panda, to assess the impacts of climate change on its distribution pattern using ensemble modeling. Our objectives are as follows: (1) identify the critical environmental variables influencing its distribution; (2) determine its potential distribution range in China under current conditions and two future periods (the 2050s and the 2090s) with three different climate scenarios (SSP126, SSP245, SSP585); (3) predict the migration trends of its habitat centroid from present to the future. This study will provide a theoretical basis for the scientific utilization, management and conservation of *F. nitida*, and hold practical significance for the protection of giant pandas and the maintenance of ecosystem stability.

## 2 Materials and method

### 2.1 Collection of distribution data

To obtain the occurrence data of *F. nitida* in China, we carried out the collection through the following pathways: (1) extensive field surveys conducted from 2019 to 2022; (2) comprehensive database search, including Chinese Virtual Herbarium Database (CVH: https://www.cvh.ac.cn/), Global Biodiversity Information Facility (GBIF; https://www.gbif.org), and National Specimen Information Infrastructure (NSII: https://www.nsii.org.cn/); (3) relevant published literatures (Zhang et al., 2019; Zhou et al., 2020; Huang et al., 2021). A total of 145 records were initially obtained, of which 51 were collected from our field surveys, 44 were retrieved from online database, and 50 were extracted from published literatures. For five records with specific locations but lacking GPS data, Baidu Maps was used to estimate their coordinate information. Data with ambiguous geographic details, duplicates, and outliers were excluded. Meanwhile, to migrate the sampling bias, we further processed the geographic distribution data using the ENMTools approach (Warren et al., 2010), with only one point retained per 30-second grid cell (1 km × 1 km). Finally, 78 reliable distribution points of *F. nitida* were confirmed (**Figure 1**; **Supplementary Table S1**).

### 2.2 Selection and processing of environmental variables

Initially, 56 environmental variables were selected in this study, including 19 bioclimate variables, 34 soil variables, and three topographic variables. Bioclimatic data were obtained from WorldClim v2.1 (https://worldclim.org/), covering the current period (1970-2000) and two future period: the 2050s (2041-2060) and 2090s (2081-2100). For future climate projections, the Beijing Climate Center Climate System Model (BCC-CSM2-MR) from the Coupled Model Intercomparison Project 6 (CMIP6) was employed, as it is well-suited for simulating bioclimate changes in China. Three Shared Socioeconomic Pathways (SSP) were selected to provide a more comprehensive understanding of the distribution pattern of *F. nitida*: SSP126, SSP245 and SSP585. These scenarios correspond to a sustainable development pathway (with global warming of 1.8°C and radiative forcing reaching 2.6 W/m² by 2100), a moderate development pathway (2.7°C and 4.5 W/m²), and a traditional high-emission pathway (4.4°C and 8.5 W/m²), respectively (IPCC, 2023). Soil variables were sourced from the Harmonized World Soil Database v2.0 (HWSD2, https://www.fao.org/soils-portal/), with D1 data extracted therefrom. Elevation data was obtained from the Geospatial Information Authority of Japan (https://globalmaps.github.io/el.html), while slope and aspect were derived from it using ArcGIS 10.7. Soil and topographic data were assumed to remain unchanged in the future. All environmental variable layers were resampled to a uniform 30 arc-second resolution and rasterized to align with the occurrence data layer in terms of boundaries, cell size, and coordinate system.

To reduce the impact of multicollinearity on prediction accuracy, we conducted a correlation analysis on the 56 environmental variables (**Supplementary Table S2**). First, we performed pre-simulation of all environmental factors in MaxEnt v.3.4.4 and excluded variables with a contribution percentage below 1% (Qiu et al., 2023b). Second, For the remaining variables, we used the Pearson method in SPSS v.23 to test correlations, when two variables exhibited a strong correlation (|r| > 0.8), the one with a higher contribution was retained. Finally, seven environmental variables were selected for subsequent ensemble modeling (**Table 1**).

**Table 1:** Environmental variables retained after processing.

Variable	Description	Unit
Bio4	Temperature seasonality	%
Bio6	Min. Temperature of coldest month	°C
Bio12	Annual precipitation	mm
Bio14	Precipitation of driest month	mm
Elevation	Elevation	m
Slope	Slope	°
WRB4	World reference base for soil resources	/

### 2.3 Construction and evaluation of the ensemble model

Firstly, we optimized two key parameters of MaxEnt—regularization multiplier (RM) and feature class (FC)—using the R package Kuenm to enhance the model’s accuracy before ensemble modeling (Cobos et al., 2019). For the RM parameter, we set a range from 0.5 to 6 with an interval of 0.5. For FC, we combined the five features of MaxEnt (linear (L), quadratic (Q), product (P), threshold (T), and hinge (H)) and generated 31 combinations. A total of 1209 candidate models were tested, and their performance was evaluated based on the Akaike Information criterion correction (AICc) and 5% Test Omission Rate (OR). Parameters with lower delta AICc and OR values were selected (Gao et al., 2024).

After parameter optimization, we first evaluated the accuracy of ten individual models—Generalized Linear Model (GLM), Generalized Boosting Model (GBM), GAM, Classification Tree Analysis (CTA), Artificial Neural Network (ANN), Surface Range Envelop (SRE), Flexible Discriminant Analysis (FDA), Multivariate Adaptive Regression Splines (MARS), RF, and optimized MaxEnt—using kappa, TSS, and AUC values. Then an optimal ensemble model was constructed from models with kappa > 0.70, TSS > 0.85, and AUC > 0.95. Through comparing the predictive accuracy of individual models and the ensemble model, we selected the latter one for subsequent prediction due to its better performance. All model construction processes were implemented in the R package Biomod2 with 10 repetitions. Key parameters were set as follows: 75% of the 78 distribution points were randomly selected as the training data, with the remaining serving as the test data; all models employed default parameters in Biomod2 except MaxEnt, which used RM and FC combinations defined by the Kuenm package. To reduce sampling bias, 1000 pseudo-absence points with 2 replications were randomly generated and included in the modeling process.

The performance of the ensemble model was evaluated using AUC, kappa and TSS. The AUC value ranges from 0 to 1, while kappa and TSS values range from -1 to 1, with higher values indicating more reliable prediction results. Common evaluation criteria are as follows: for AUC, values between 0.50 and 0.70 indicate poor performance, 0.70 < AUC ≤ 0.80 indicate fair performance, 0.80 < AUC ≤ 0.90 indicate good performance, and values above 0.90 represent excellent performance; For kappa and TSS, values between 0.40 to 0.50 indicate poor performance, 0.50 < kappa/TSS ≤ 0.70 indicate fair performance, 0.70 < kappa/TSS ≤ 0.85 indicate good performance, and values above 0.85 represent excellent performance.

### 2.4 Analysis of key environmental factors

To identify the critical environmental variables influenced the distribution of *F. nitida*, we calculated the weights of the selected environmental variables in each model and selected the top three ones. Response curves of the significant environmental factors were plotted using “jackknife” method in the optimized MaxEnt model.

### 2.5 Analysis of suitable habitat patterns

The suitable areas for *F. nitida* under both current and future climate scenarios were simulated using the ensemble model. Projection outputs were imported into ArcGIS 10.7 and reclassified based on the ecological criteria of *Fargesia denudate*, a congeneric species distributed in a similar region (Hu and He, 2020). These areas were divided into four categories: unsuitable areas (< 0.25), low suitable areas (0.25-0.50), moderately suitable areas (0.50-0.75), and highly suitable areas (> 0.75). The area of suitable habitats for *F. nitida* under each climate scenario was calculated using ArcGIS 10.7. Binary maps were then generated to determine the changing pattern of habitats under different emission scenarios, with contraction, expansion, and unchanged areas calculated using the “distribution changes between binary SDMs” tool from the SDM Toolbox v2.5 (Brown, 2014). Finally, the “centroid changes clines” tool in ArcGIS was used to identify the shift direction of the distribution centroid.

## 3 Results

### 3.1 Model evaluation and key environmental factors

**Figure 2:**
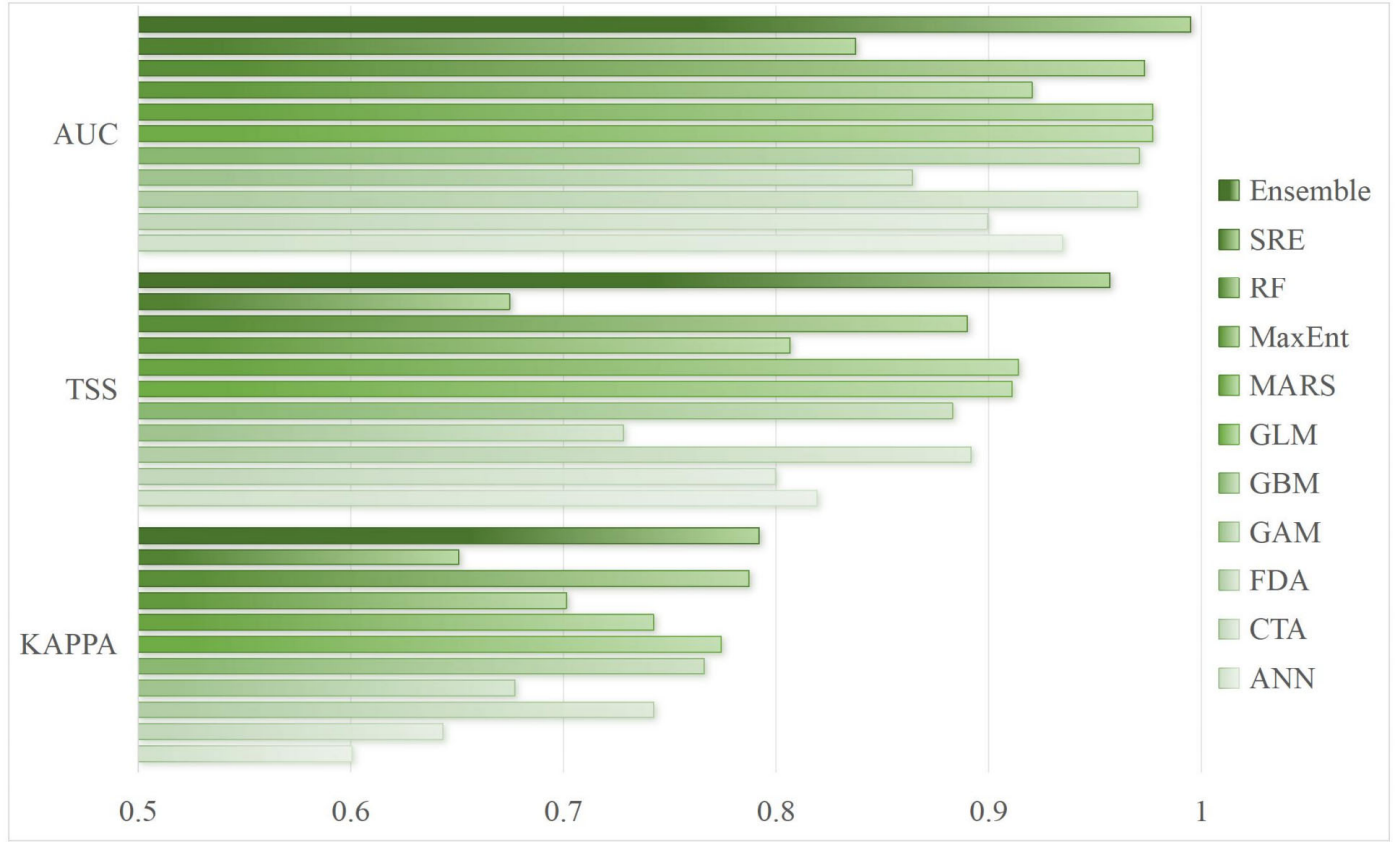
Model evaluation of the ensemble model and ten individual models based on kappa, TSS and AUC.

Using the Kunem package, we optimized the two key parameters of MaxEnt based on 78 distribution points of *F. nitida* and seven environmental factors. The delta AICc and OR values of the default parameters (RM = 1, FC = LQHPT) were 103.10 and 0.12, respectively, whereas these values decreased substantially to 0 and 0.04 under the parameter configuration of RM = 0.1 and FC = LQ. Hence, this optimized configuration was selected for subsequent MaxEnt analyses. Meanwhile, comparison of the evaluation metrics across all models revealed that the ensemble model achieved the highest kappa, TSS and AUC values, reaching 0.753, 0.957 and 0.995, respectively (**Figure 2**), indicating optimal prediction accuracy. Consequently, the ensemble model was chosen for projecting the distribution pattern of *F. nitida*.

The importance values of bioclimatic variables across all individual models revealed that temperature seasonality (bio4) and min temperature of coldest month (bio6) had the greatest impact on the geographic distribution pattern of *F. nitida*, followed by elevation, precipitation of driest month (bio14), and annual precipitation (bio12) (**Table 2**). Notably, in the ANN model, bio12 emerges as the most important factor, indicating that precipitation may also play an important role in shaping the distribution pattern of *F. nitida*. In contrast, the effects of soil type (WRB4) and slope were negligible.

**Table 2:** Importance values of environmental variables influencing the distribution of *F. nitida*.

Variable	GLM	GBM	GAM	CTA	ANN	SRE	FDA	MARS	RF	MaxEnt
Bio4	**0.82**	**0.49**	**0.81**	**0.54**	0.32	**0.45**	**0.77**	**0.68**	**0.22**	**0.97**
Bio6	**0.56**	**0.45**	**0.65**	**0.62**	**0.46**	**0.51**	0.21	0.27	**0.28**	**0.71**
Bio12	0.13	0.02	0.46	0.06	**0.73**	**0.40**	0.05	0.16	**0.12**	0.34
Bio14	**0.45**	**0.16**	0.41	**0.38**	0.32	0.20	**0.24**	**0.35**	0.09	0.56
Elevation	0.38	0.07	**0.52**	0.09	**0.44**	0.29	**0.39**	**0.40**	0.08	**0.66**
Slope	0.01	0.00	0.15	0.00	0.03	0.18	0.00	0.00	0.02	0.08
WRB4	0.08	0.01	0.30	0.01	0.09	0.27	0.00	0.02	0.03	0.07

Bold values represent the top three import environmental variables in each model.

**Figure 3:**
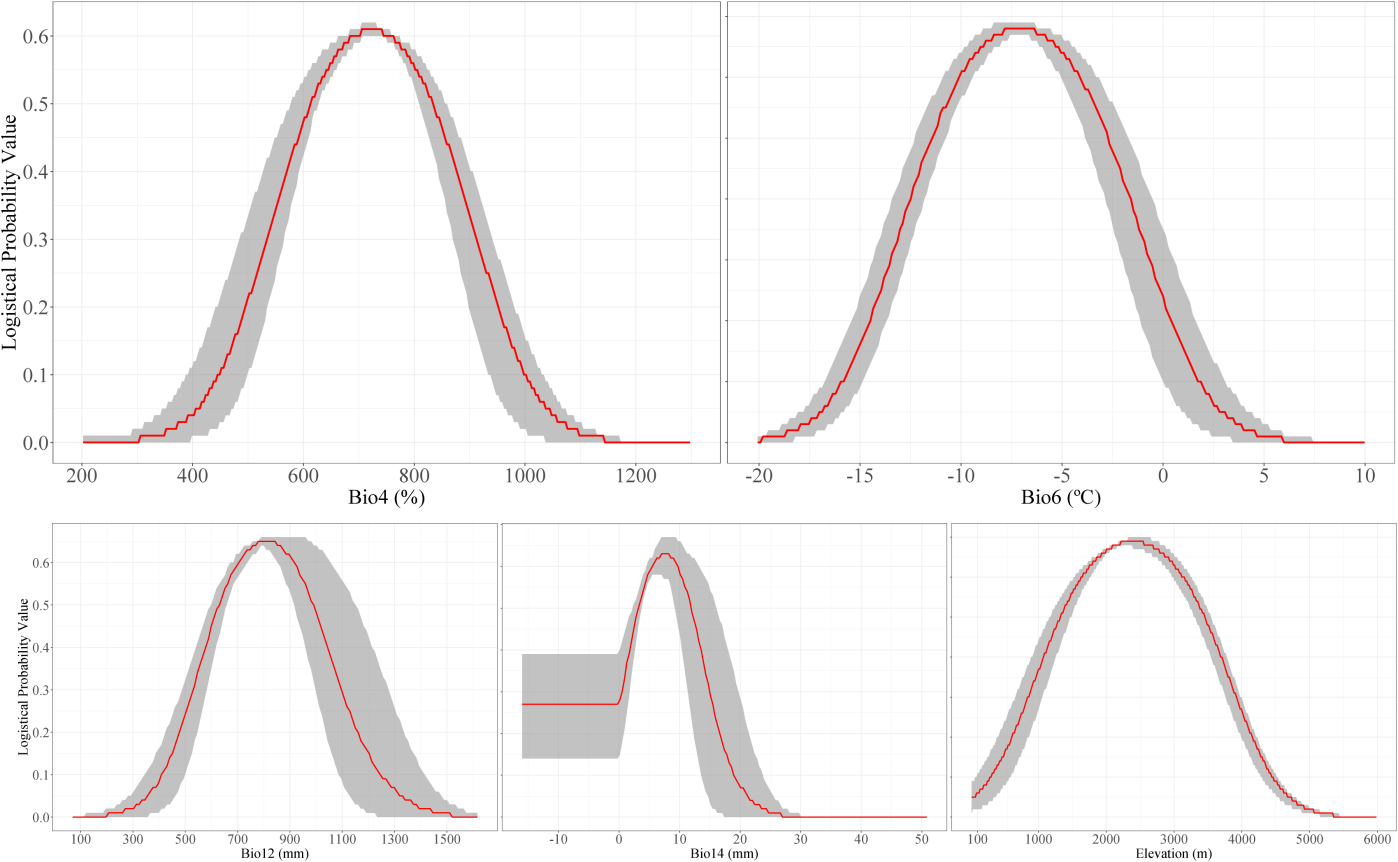
Response curve for the main environmental variables, including temperature seasonality bio4), min. temperature of coldest month (bio6), annual precipitation (bio12), precipitation of driest month (bio14), and elevation.

Response curves can illustrate the quantitative relationship between the logistic occurrence probability and the appropriate range of environmental variables. Generally, a probability exceeding 0.5 indicates that such an environment is conducive to the growth of bamboos. As shown in **Figure 3**, *F. nitida* exhibits favorable growth and occurrence when temperature seasonality ranges from 613% to 834%, min temperature of coldest month from -11.43 °C to -2.79 °C, elevation from 1324 m to 3457 m, annual precipitation from 630 mm to 991 mm, and precipitation of driest month from 3.20 mm to 11.65 mm.

### 3.2 Current potential suitable habitat of *F. nitida*

**Figure 4:**
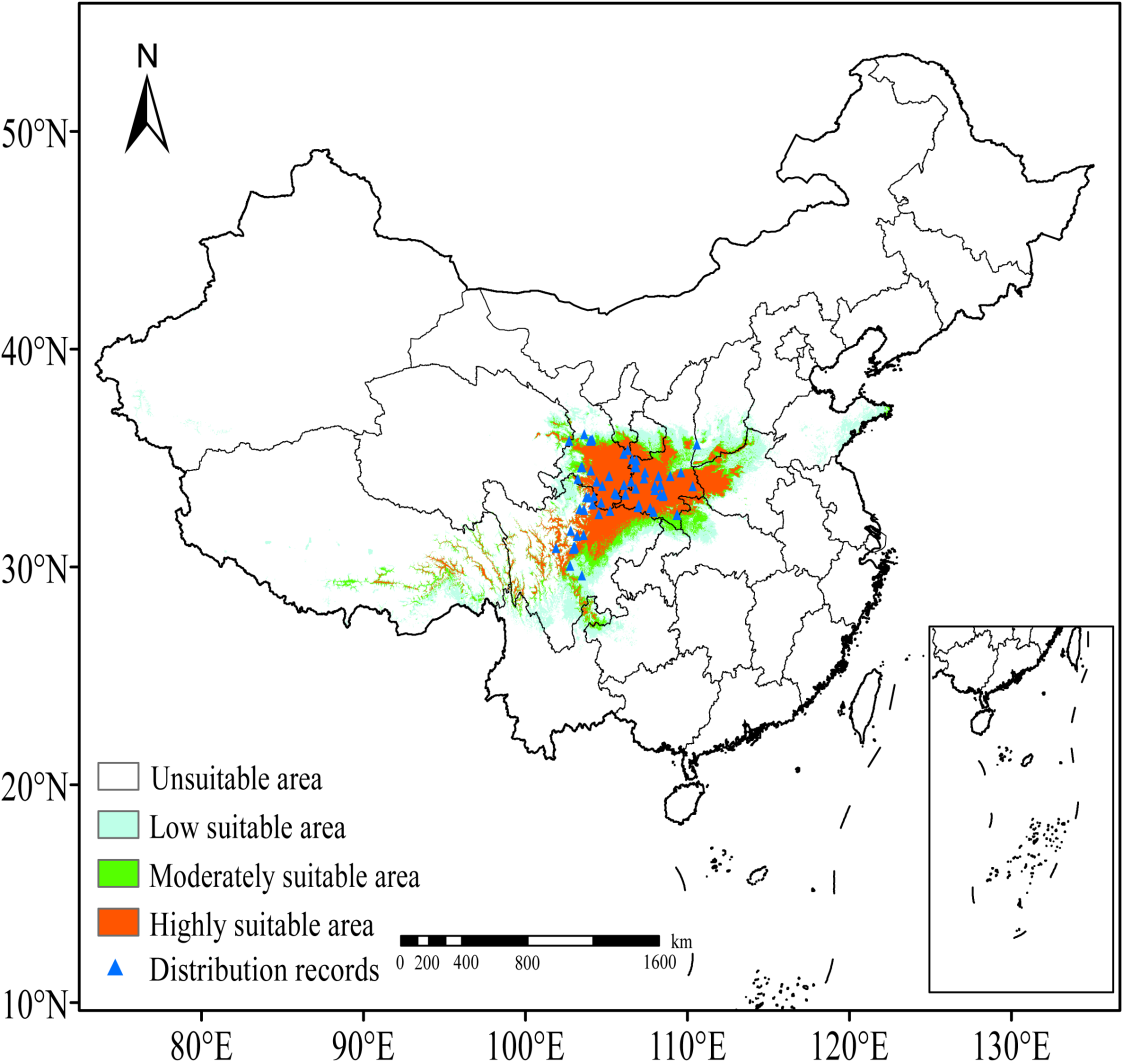
Potential geographical distribution of Fargesia nitida at current condition.

As determined by the ensemble model, the current suitable habitat for *F. nitida* is mainly located in the HDM region and its adjacent areas, ranging from 102° E – 113° E, 29° N – 36° N. The total suitable area is 83.10 × 10^4^ km^2^, accounting for 8.69% of China’s land area. However, within this total area, the highly suitable area constitutes a relatively small proportion (only 39.51%), covering 32.84 × 10^4^ km². Highly suitable habitats are mainly concentrated in Sichuan, Gansu, Shaanxi, and Henan, and almost completely overlap with the actual distribution points (**Figure 4**). Moderately suitable areas are mainly distributed around the highly suitable habitats, with sporadic distribution in Hubei, Yunnan and Xizang, covering 16.18 × 10^4^ km², which accounts for 19.47% of the total suitable area. Low suitable areas, where no sampling points have been recorded, are primarily located in southern Sichuan, Henan, Shandong, and Shanxi, with scattered occurrences in Yunnan, Guizhou, Xizang and Xinjiang.

### 3.3 Dynamic changes in suitable habitats under different climate scenarios

**Figure 5:**
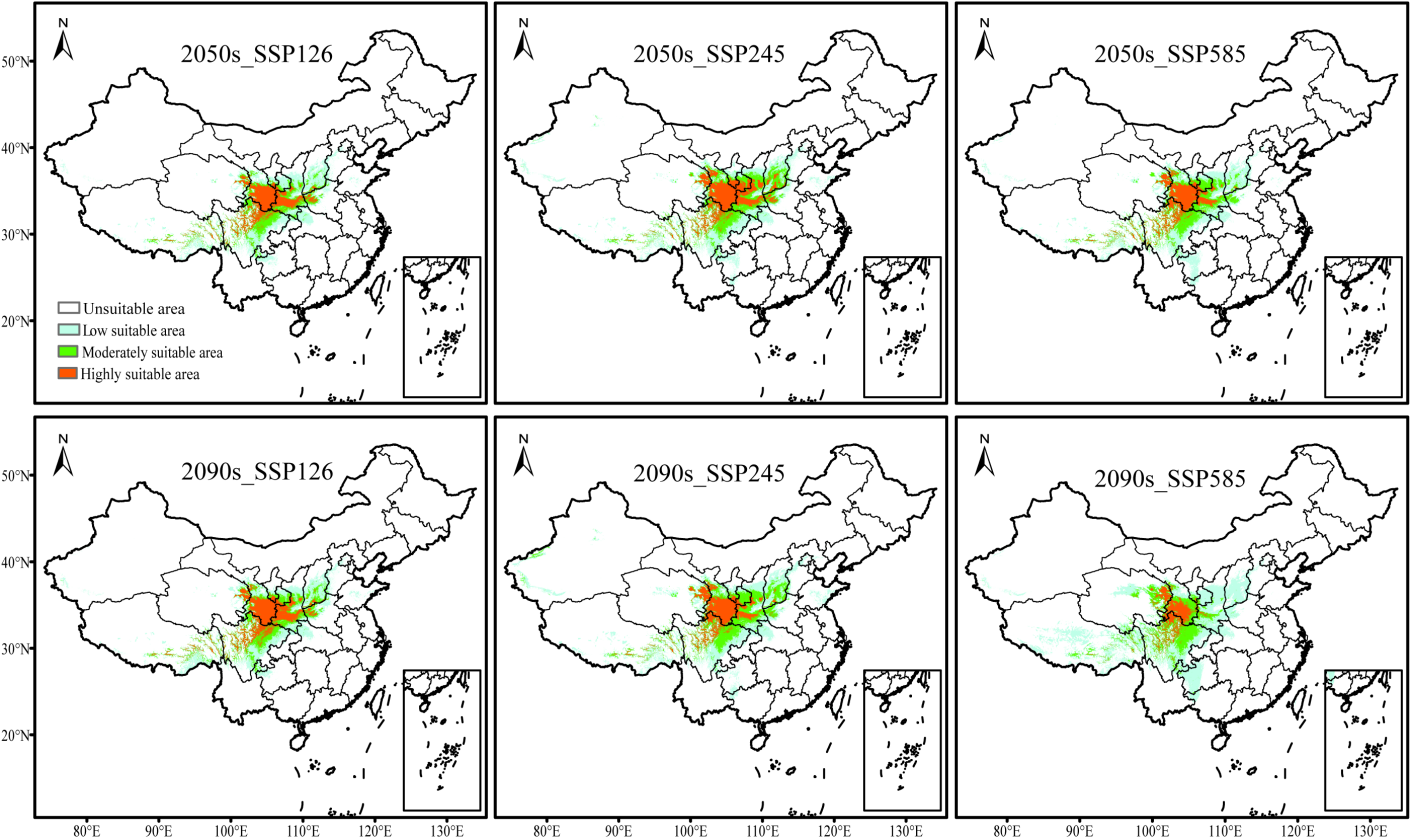
Potential suitable areas for *Fargesia nitida* under future climate scenarios. 2050s represents 2040–2060s period and 2090s represents 2080–2100 s period. SSP126, SSP245, and SSP585 represents different shared socioeconomic pathways.

**Figure 6:**
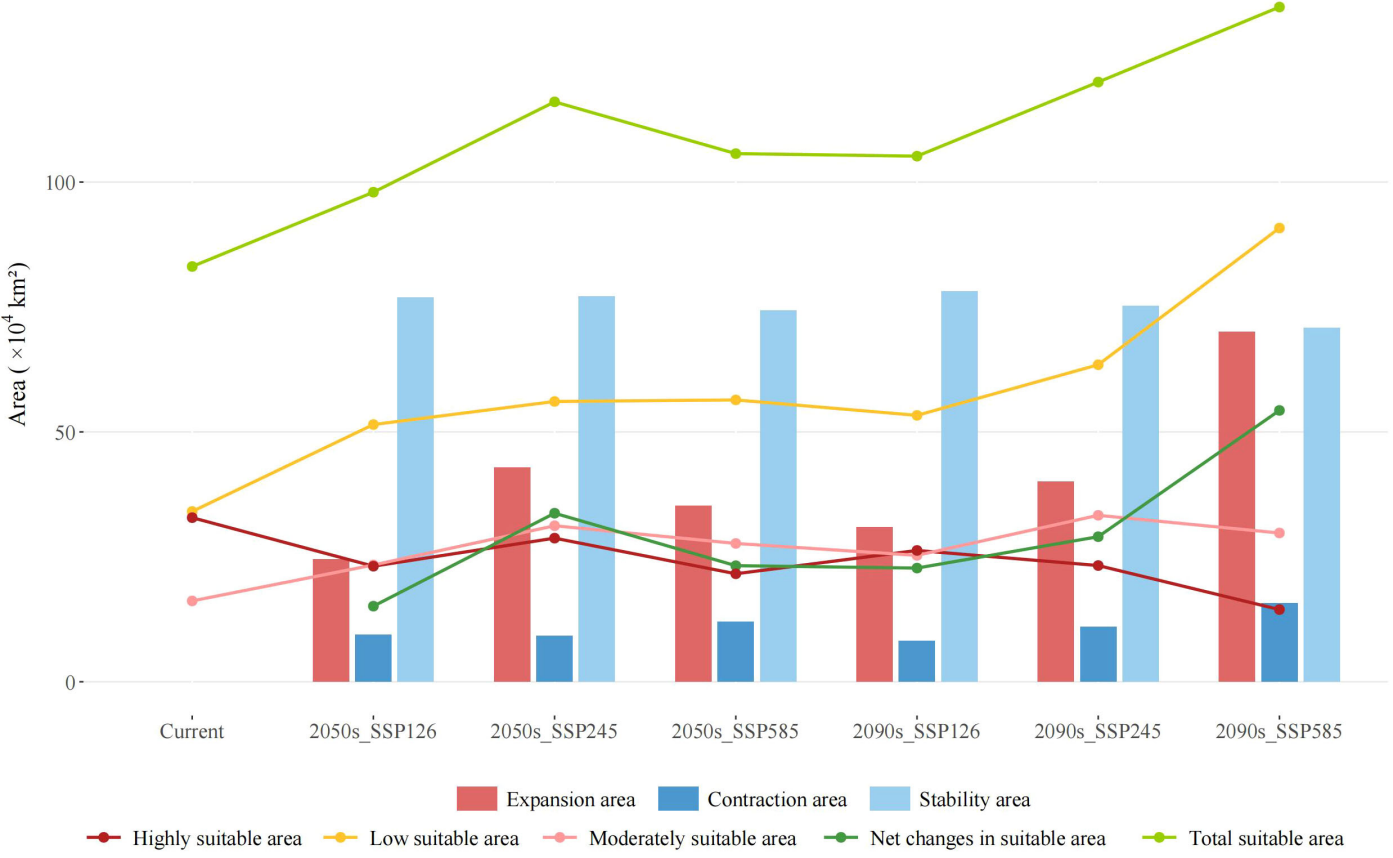
Variation in suitable area for *Fargesia nitida* under different climate scenarios. SSP126, SSP245, and SSP585 represents different shared socioeconomic pathways.

With ongoing environmental changes, the suitable habitat of *F. nitida* remains concentrated in the HDM region and its adjacent areas, but the total suitable area exhibits an expanding trend across climate scenarios (**Figures 5**, **6**; **Supplementary Figure S1**). Under the SSP126 scenario, the total suitable area will increase to 97.07 × 10^4^ km² by the 2050s period and then decrease to 85.18× 10^4^ km² by the 2090s, representing a 17.89% and 2.5% increase compared to the current scenario, respectively. Under the SSP245 scenario, the total suitable area will continue to increase, reaching 116.05 × 10^4^ km² by the 2050s and 120.01 × 10^4^ km² by the 2090s, with respective growth ratio of 36.95% and 44.41%. Under the SSP585 scenario, an even greater increase in the total suitable area is projected, reaching 112.73 × 10^4^ km² by the 2050s and 135.02 × 10^4^ km² by the 2090s, with the growth rate reaching 62.48% in the 2090s.

Comparison suitability zones (**Figure 6**; **Supplementary Figure S1**) revealed that the total suitable area expansion is primarily driven by increased low suitable area, supplemented by a slight growth in the moderate suitable areas. However, the highly suitable areas—the primarily distribution zone of *F.nitida*—decline consistent across all scenarios, indicating that the actually suitable area for this bamboo species is decreasing. While the expanded area exceeded the contracted area overall, the extent of expansion and contraction varies across different climate scenarios, which could lead to changes in the distribution pattern of *F. nitida*. For example, both the greatest expansion and contraction occurred at the SSP585 scenario in the 2090s, whereas the SSP245 scenario in the 2090s experiences the largest increase in the moderately suitable area.

**Figure 7:**
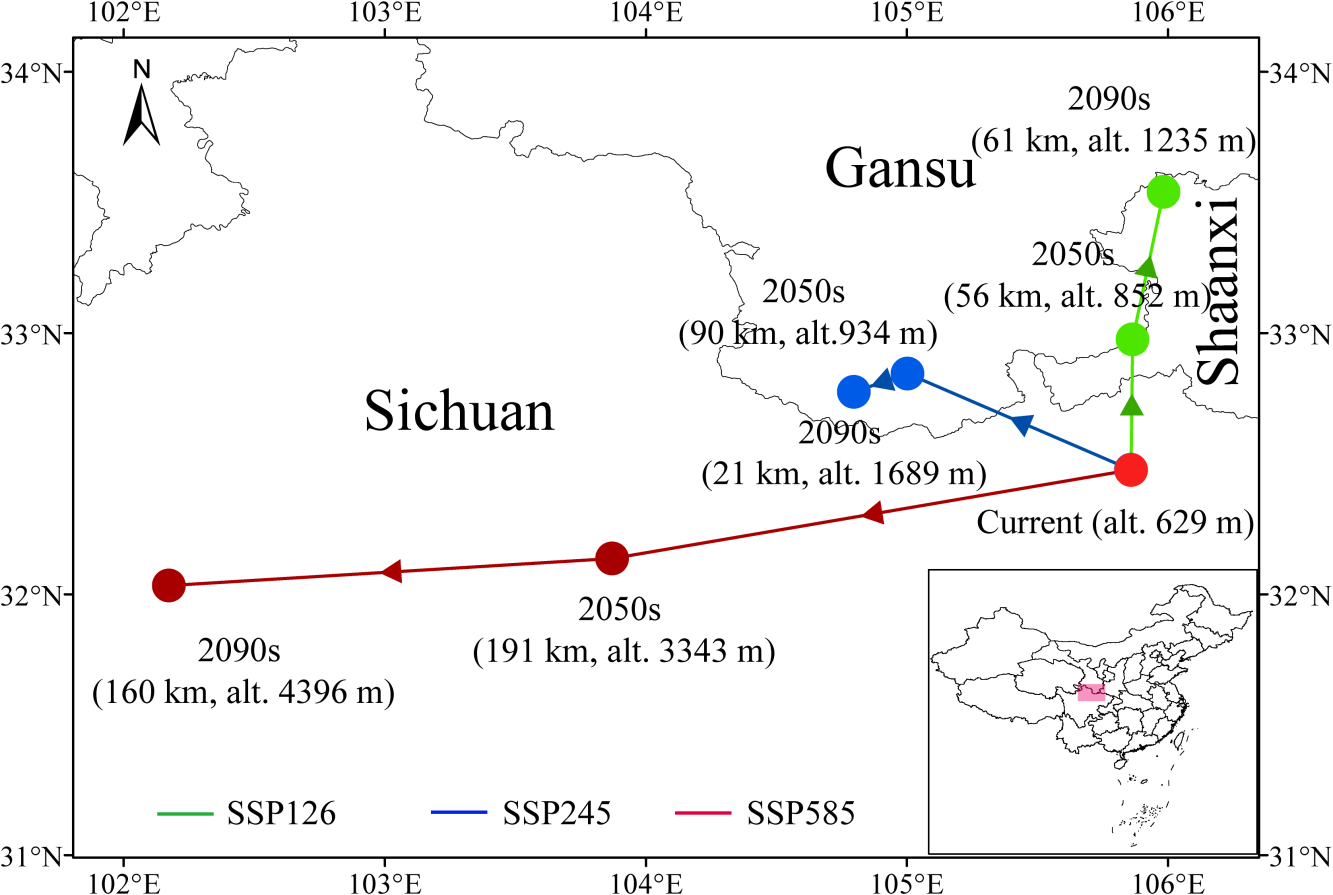
The migration trajectory of distribution centroids of *Fargesia nitida* under different climate scenarios. SSP126, SSP245, and SSP585 represents different shared socioeconomic pathways.

Along with changes in suitable habitats, the spatial distribution centroid of *F. nitida* is projected to shift under future climate scenarios, as shown in **Figure 7**. Currently, the distribution centroid of *F. nitida* is situated at the northern border of Sichuan province (105°51′32.02″ E, 32°28′32.35″ N), and it will migrate to different directions under various emission scenarios. For instance, under the sustainable development scenario (SSP126), the species shift northward by 56 km in the 2050s and 61 km in the 2090s. Under the moderate development scenario (SSP245), *F. nitida* first move 90 km northwestward in the 2050s, then shift slightly southwestward by 21 km in the 2090s. Such northward or northwestward shift in the low-emission scenarios (SSP126 and SSP245) is primarily driven by the expansion of low-moderate suitable habitats in northern Gansu, Shaanxi, and central Shanxi (expansion ca. 50%) and the contraction of highly suitable area in northern Sichuan (20%-30% loss) (**Figure 5**; **Supplementary Figure S1**). Under the traditional high-emission scenario (SSP585), the species will continuously migrate southwestward, with distances of 191 km in the 2050s and 160 km in the 2090s. This southwestward shift is dominated by the contraction of core highly suitable area in Sichuan and Shaanxi (with a loss of up to 56%) and the expansion of marginal habitats toward Xizang, Qinghai and western Sichuan. Concurrently, the altitude of the distribution centroid will gradually increases (**Figure 7**), particularly under the SSP585 scenario. As the species’ range shift to the Qinghai-Tibet Plateau, the altitude of its distribution centroid will rise to 3343 m by the 2050s and reach 4396 m by the 2090s. These findings demonstrate that different climate scenarios exert a significant influence on the distribution pattern of *F. nitida*.

## 4 Discussion

### 4.1 Optimization and limitation of SDMs

In the last thirty years, SDMs have been widely applied in conservation biology, ecology, and evolutionary biology to guide species conservation and management efforts (Wudu et al., 2023; Zhang et al., 2023). However, challenges and caveats have been pointed out regarding their predictions of species’ future distribution (Peterson et al., 2018), and parameter setting and model selection are critical to improve the accuracy of species distribution modeling (Bellard et al., 2012; Gao et al., 2024). Among SDMs, MaxEnt has been extensively employed to predict the distributions of endangered species, alien species and pests for its better performance than most other models (Elith et al., 2006). Furthermore, it yield more accurate predictions when using optimized configurations than those using default settings (Fang et al., 2022; Zhao et al., 2024). However, different models may produce biased predictions for their varied algorithm principles and assumptions. To address this limitation and improve prediction accuracy, we integrated the optimized MaxEnt model with biomod2 to construct an ensemble model. By combing models with kappa > 0.70, TSS > 0.85, and AUC > 0.95, the ensemble model achieved higher accuracy than both the optimized MaxEnt model and other individual model across all evaluation metrics (**Figure 2**). Both TSS and AUC values of the ensemble model reached the excellent level (TSS: 0.907; AUC: 0.988), indicating that our ensemble model produces credible simulation results and can reliably predict shifts in the distribution pattern of *F. nitida*.

Notably, several limitations may affect the interpretation of results in our study. First, the spatial scale may introduce uncertainties into SDMs outputs. Researches on geographic distribution of alien ornamental plants have revealed that predictions at the regional scale can yield a broader suitable range compared to those at the national scale (Oduor et al., 2023). Therefore, our simulation of the potential distribution area at China’s national scale may underestimate the suitable range in localized microhabitats according to the geographical scale-dependent perspective (Levin, 1992). Second, although we used ENMTools to reduce sampling bias (retaining one point per 1 km × 1 km grid), the 78 final occurrence records still have limited coverage of remote areas in the western HDMs. Such under-sampling and uneven sampling may lead to biased estimation of suitable habitats in these regions, which is consistent with the conclusions summarized by Santini et al. (2021). Third, physiological traits usually affect the climate niche breath of plants (Yan et al., 2025). Specifically, *F. nitida* has unique life history traits—short rhizome necks (10–13 cm) and long flowering cycles (up to 109 years)—which greatly restrict its dispersal capacity under rapid climate change. This is contradictory with the model assumption in our study that the species can migrate freely to newly suitable habitat. Hence, the actual distribution area may be narrower than the predicted one. Future studies could address these limitations by adjusting research scale, expanding field surveys, and incorporating physiological factors into model simulations.

### 4.2 Key environmental factors influencing the distribution of *F. nitida*

The geographic distribution pattern of *F. nitida* is primarily determined by temperature-related factors (bio4 and bio6), followed by elevation and precipitation-related factors (bio14 and bio12). This finding contrasts with several studies predicting bamboos’ distribution, which identified precipitation as the primary determinant of their distribution, with temperature playing a secondary role (Li et al., 2019; Zhao et al., 2023). Our results confirm that temperature is the dominant factor shaping the distribution of *F. nitida*—a pattern consistent with the modeling results of *Fargesia denudata*, a congeneric species also endemic to the HDM region (Hu and He, 2020). The dominance of temperature in shaping *F. nitida*’s distribution is closely tied to the species’ adaptation to alpine environments and its unique life-history traits. Specially, suitable seasonality (bio4: 613%-834%) is conducive to the emergence of bamboo shoots and the formation of lignification, which are the key processes for bamboos’ growth and cold resistance. Meanwhile, optimal minimum temperature in the coldest month (bio6: -11.43 °C to -2.97 °C) ensure winter survival by preventing freeze damage to rhizomes, the core organ for bamboos’ clonal reproduction (Li et al., 2019). This temperature-driven distribution pattern is not unique to *F. nitida*, researches on *Incarvillea* in the Himalayan region (Rana et al., 2021)and major tree species in Southwest China (Ma et al., 2022) have similarly found that temperature-dependent variables exert a more dominant influence on species distribution than precipitation-related factors. This suggests that temperature may play a more critical role in determining the habitat suitability for plants distributed in alpine and southwestern China mountains. However, additional studies involving more taxa are needed to validate this hypothesis.

Additionally, elevation plays an important role in shaping the richness pattern of montane plants (Hu and He, 2020; Qiu et al., 2023a). *F. nitida*, which is mainly distributed in the subalpine zone at an altitude of 2450–3200 m, is also influenced by elevation in terms of its distribution. Our study revealed that, in response to climate change, this bamboo species will migrate to northwest with higher altitude and may reach an upper limit of 3457 m. This upward shift could facilitate its survival under future climatic conditions, just as has been observed in *Bergenia*, *Primula* and plants distributed in Mt. Gongga etc (He et al., 2019; Zu et al., 2021; Qiu et al., 2023a).

Notably, soil parameters (WRB4) and slope exert negligible impacts on *F. nitida*’s distribution (**Table 2**). This species can adapt to both acidic and neutral soil types across the HDM region by regulating nutrient availability in its rhizosphere—an adaptive trait that alleviates soil-related constraints on its range (Guo et al., 2023). For slope, our field observations confirm that *F. nitida*’s dense caespitose rhizomes (**Figure 1C**) effectively stabilize its growth even on steep terrain (slope >30°), reducing its dependence on specific slope conditions and explaining why slope has a weak predictive power for its distribution.

### 4.3 Geographic distribution changes of *F. nitida*

Climate change significantly influences plant growth and distribution patterns. Many plant species have been observed migrating toward higher altitudes and latitudes to cope with increasing environmental pressures (Yang et al., 2024; Ye et al., 2024). *F. nitida* adopts a similar adaptive strategy, but the migration trajectory of its distribution centroid varies across climate scenarios. This divergence is closely tied to the intensity of climate warming and the species’ niche requirement. Under the low-emission scenarios (SSP126 and SSP245), *F. nitida* gradually shifts northward to higher-latitude regions—where temperature conditions align with its niche requirements (**Figure 3**). This pattern is consistent with the migration behavior of bamboo species *Chimonobambusa tumidissinoda* (Wang et al., 2024). Compared to the current centroid location, the migration distance under these scenarios remains relatively limited (approximately 100 km). This suggests that the species’ core habitats still maintain partial suitability, reducing the urgency of large-scale range shifts, consistent with observations in *Alpinia officinarum*, which exhibit distribution area increase and modest northward migration under low-emission scenarios (Kang et al., 2025). In contrast, under the high-emission scenario (SSP585), *F. nitida* exhibits a distinct southwestward shift—with migration distances of 191 km in the 2050s and 160 km in the 2090s—and a continuous increase in centroid altitude. This direction change stems from the severe loss of core highly suitable areas (up to 56% contraction in Sichuan and Shaanxi) resulting from extreme temperature increase (4.4 °C by 2100; IPCC, 2023). Such thermal conditions beyond the tolerance of *F. nitida*, particularly threatening rhizome survival of bamboos (Li et al., 2019). As a result, the species is forced to migrate toward the Qinghai-Tibet Plateau, where higher altitudes (rising to 3343 m in the 2050s and 4396 m in the 2090s) provide cooler microclimates that match its temperature niche. This high-altitude migration mirrors trends in other alpine taxa of the Hengduan Mountains, such as *Bergenia* and *Primula*, which shift upward to avoid heat stress under extreme climate scenarios (Qiu et al., 2023a, b). However, *F. nitida*’s limited dispersal capacity raises concerns about its ability to colonized these new high-altitude habitats. Ye et al. (2019) noted that alpine bamboos with low dispersal rates struggle to track rapid climate shifts, and our projections suggest this risk is amplified under SSP585, where suitable habitat in the Plateau’s remote areas may remain underutilized.

From a conservation perspective, the divergent migration patterns highlight scenario-dependent vulnerability. Although the total suitable area expands across all scenarios, the consistent contraction of highly suitable habitats (the most representative of the species’ actual growth conditions; **Figure 6**) indicates underlying risks. Particularly under the high-emission scenario in the 2090s, the highly suitable area will decrease sharply to 14.45 × 10^4^ km², a reduction of over 56%, and the distribution centroid will shift to an elevation potentially beyond the dispersal ability of bamboos. Given *F. nitida*’s limited diffusion capacity and relatively recent evolutionary origin (Ye et al., 2019), its future survival prospects are concerning. This may also impose high pressure on the survival of giant pandas, which rely on it for approximately 90% of their diet. Targeted conservation measures, such as the implementation of long-term monitoring of bamboo dynamics, particularly flowering periods, the establishment of habitat corridors to prevent habitat fragmentation, and the facilitation of assisted migration to high-elevation refugia under severe emission scenarios, should be implemented to safeguard *F. nitida* populations and maintain the stability of the giant panda habitats.

## 5 Conclusion

In this study, an ensemble modeling approach was employed to evaluate the impacts of climate change on the distribution pattern of *F. nitida*, a primary food source for giant pandas and a key understory alpine bamboo species. The results revealed that temperature-related variables, particularly temperature seasonality (bio4) and minimum temperature of the coldest month (bio6), are the most critical drivers determining its distribution, followed by elevation and precipitation factors. The current total suitable habitat of *F. nitida* is estimated at 83.10 × 10^4^ km², primarily concentrated in the HDM region and its adjacent areas (102° E – 113° E, 29° N – 36° N). Highly suitable areas, accounting for 39.51% of the total suitable area, are predominantly located in Sichuan, Gansu, Shaanxi, and Henan. These highly suitable areas encompass the species’ observed distributions points and are more representative of the conditions suitable for *F. nitida*. Under future climate scenarios, while the total suitable area shows an expanding trend, the extent of highly suitable areas consistently declines. For instance, under the SSP585 scenario, the total suitable area increased by 62.48% by the 2090s, whereas highly suitable areas decrease by more than 56%. The current distribution centroid, located in northern Sichuan, exhibits consistent migration to higher elevations and scenario-specific directional changes: a northward shift (high latitude) under low emission scenarios (SSP126 and SSP245), and a southwestward shift (high altitude) under high-emission scenario. These findings highlight the vulnerability of *F. nitida* to climate change, especially under high-emission scenarios, and provide a scientific basis for its conservation, the protection of giant panda, and the management of subalpine ecosystem.

## Data Availability

The raw data supporting the conclusions of this article will be made available by the authors, without undue reservation.

## Supplementary material

Supplementary Table 1 Distribution points of *Fargesia nitida* used in this study.
